# Performing womanhood in schools: a feminist phenomenological investigation of female teachers’ psycho-social health experiences and implications for educational leadership in Turkey

**DOI:** 10.3389/fpsyg.2025.1692323

**Published:** 2025-12-03

**Authors:** Engin İŞ

**Affiliations:** Department of Child Development, Faculty of Health Sciences, Mardin Artuklu University, Mardin, Türkiye

**Keywords:** feminist phenomenology, psycho-social health, gender performativity, work–family conflict, emotional labor, educational leadership, educational management

## Abstract

This study employs a feminist phenomenological approach to examine the psycho-social health experiences of female teachers in Turkey as they navigate the demands of professional and domestic life. Drawing on semi-structured interviews with 28 teachers across different school levels, the study explores how participants internalize, negotiate, and perform socially constructed gender roles in both public (school) and private (home) spheres. Thematic analysis identified key challenges, including role conflict, emotional labor, burnout, and guilt, all shaped by dominant gender norms and structural inequalities. Grounded in feminist theories—gender performativity (Butler), hegemonic masculinity (Connell), and intersectionality (Crenshaw)—the study demonstrates the embodied impacts of gendered oppression on women’s mental health. Participants’ coping strategies, including resilience and social support, are interpreted as both survival mechanisms and subtle forms of resistance. The findings underscore the urgent need for gender-sensitive institutional policies, flexible working arrangements, and psychosocial support programs to promote teachers’ well-being and equality in the education system.

## Introduction

Teaching, as a profession, encompasses far more than the transmission of academic knowledge—it is deeply embedded within the reproduction of social norms, values, and gendered expectations (Apple, 2004). In Turkey, teaching has long been regarded as a “respectable” and “appropriate” occupation for women, contributing to the feminization of the profession and increasing female representation in public employment (Keskin, 2022). This gendered perception significantly influences both career choices and the subsequent formation of professional identity among female teachers (Soylu and Esen, 2022). According to the Ministry of National Education (Turkey) (2024), women constitute nearly 60% of the teaching workforce, underscoring the central role of female educators in the Turkish education system (Turkish Statistical Institute, 2024). Despite this strong presence, women’s professional lives are shaped by enduring gender norms that extend their responsibilities beyond the classroom. Female teachers are expected to maintain professional excellence while simultaneously fulfilling domestic and caregiving duties—a dual burden that has been well documented globally (Hochschild, 1989; OECD, 2021; UNESCO, 2022). This invisible labor, which encompasses childcare, eldercare, and household management, intensifies psychosocial strain and challenges women’s ability to sustain a healthy work–family balance, contributing to stress, burnout, and guilt (Maslach and Leiter, 2016; Bilodeau et al., 2023).

**Figure 1:**
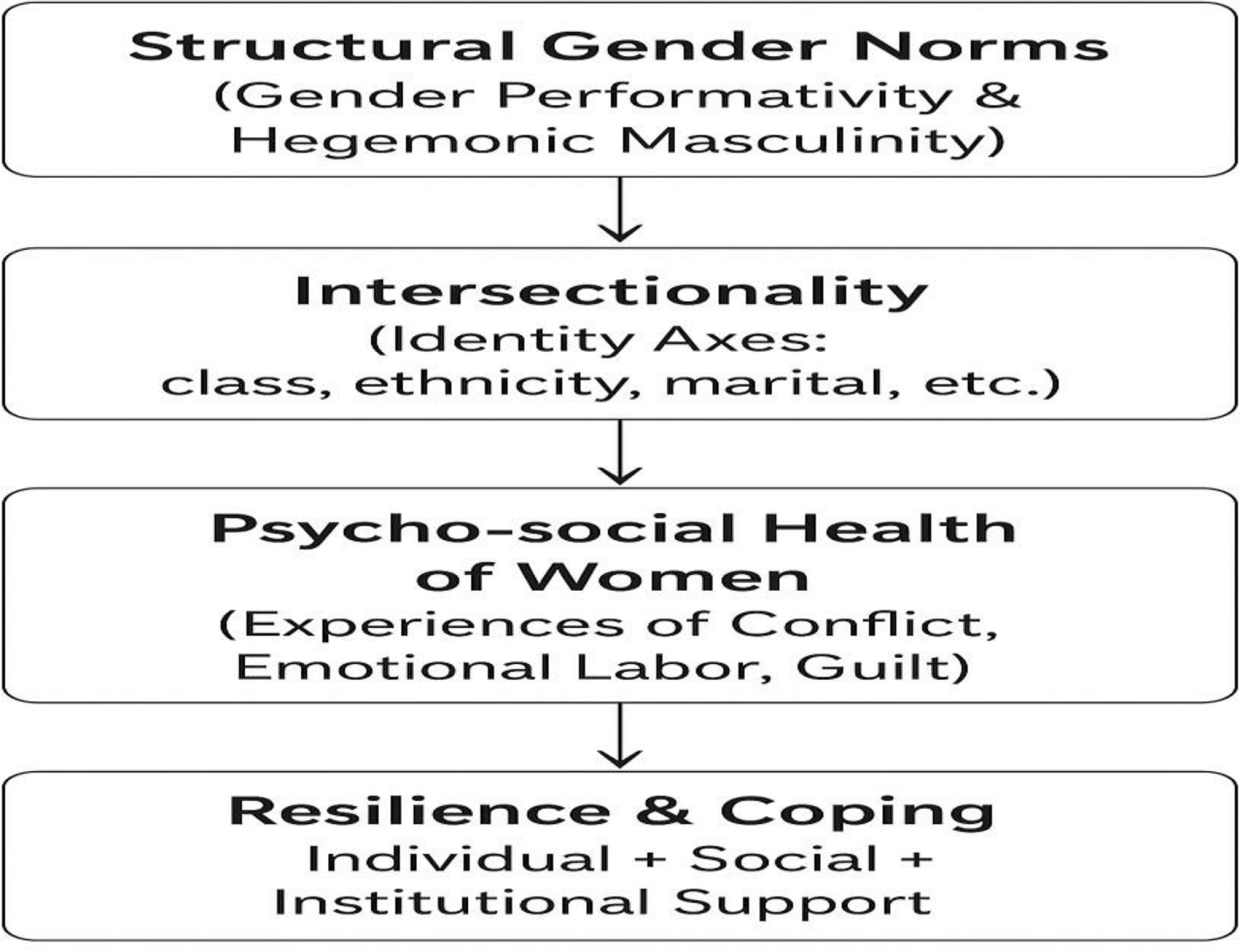
Conceptual framework of women’s psycho-social health: from structural gender norms to resilience and coping.

Although international research highlights the mitigating effects of supportive institutional measures (Beauregard and Henry, 2009; Lahelma et al., 2014; Kimura, 2010), such mechanisms remain underdeveloped in Turkey. Evidence indicates that female teachers with young children experience particularly high levels of work–family conflict, emotional exhaustion, and psycho-social pressure (OECD, 2021; Keskin, 2022). Professions requiring high emotional labor, such as teaching, further amplify these challenges, often resulting in feelings of inadequacy, stress, and persistent tension between professional and private roles (Allen et al., 2000; Kinman et al., 2011). Gender-sensitive working conditions, as recommended by the International Labour Organization (2018), remain insufficiently implemented, highlighting structural barriers that exacerbate female teachers’ psychosocial burdens. Despite abundant quantitative research on work–family balance, qualitative studies examining female teachers’ lived experiences, emotional processes, and coping mechanisms are limited, leaving a critical gap in understanding how gender norms and institutional structures interact with personal resilience (Figure 1).

In response to this gap, the present study aims to explore how female teachers in Turkey experience, negotiate, and cope with the tensions between their professional and domestic roles. Grounded in feminist phenomenology, the research seeks to illuminate the interplay between structural gender norms, institutional policies, and individual resilience strategies, revealing both the challenges and the adaptive responses of female educators. Understanding these dynamics is essential, as failure to address them may perpetuate burnout, reinforce gender-based inequalities in education, and hinder the development of equitable, supportive working environments. By highlighting individual experiences alongside structural constraints, this study intends to contribute to gender-sensitive policies, school-based interventions, and professional practices that enhance female teachers’ well-being and professional sustainability, making the often-invisible labor visible and psycho-social costs borne by women in education (Hooks, 2000; Hochschild, 1989; UNESCO, 2022).

## Theoretical framework

Understanding the psycho-social health experiences of female teachers caught between work and family life requires a multilayered analysis encompassing individual, social, cultural, and institutional dimensions. To capture these complexities, this study adopts an integrative framework combining gender theories, intersectionality, and psychological resilience theory. These perspectives function as complementary lenses that illuminate both structural and individual aspects of women teachers’ experiences in Turkey (Table 1).

1. Gender theories and Turkish socio-cultural norms

**Table 1:** Distribution of participants according to demographic characteristics.

Characteristic	Subcategories	Frequency
School type	Preschool	7
	Primary school	7
	Middle school	7
	Secondary school	7
Age range	26–30	6
	31–35	8
	36–40	6
	41–50	8
Marital status	Married	20
	Single	8
Professional tenure	3–7 years	8
	8–14 years	10
	15 years and above	10

Gender theories emphasize that femininity and masculinity are socially constructed rather than biologically determined. Judith Butler’s (1990) performativity approach frames gender identities as continuously reproduced through discourses and practices. In the Turkish context, female teachers are expected to fulfill dual roles—as “ideal workers” in professional settings and “ideal mothers and spouses” in private life—reflecting entrenched gender norms. Connell’s (2013) notion of hegemonic masculinity situates schools as institutional sites where normative gender hierarchies are maintained, affecting both female and male teachers’ behaviors. These theories provide a structural lens to understand how institutional and societal expectations shape daily realities of Turkish women educators.

Furthermore, Bell hooks' (2000) feminist theory, which centers the experiences of marginalized voices and critiques patriarchal systems, provides a crucial lens for understanding how female teachers’ daily struggles are not merely personal failures but manifestations of structural oppression. Her concept of moving women’s experiences ‘from margin to center’ aligns with this study’s commitment to treating women’s lived experiences as legitimate sources of knowledge, challenging the patriarchal norms that normalize their double burden.

2. Intersectionality in the Turkish context

Intersectionality (Crenshaw, 1989) highlights that women’s experiences are shaped by the interplay of multiple identity axes, such as class, ethnicity, marital status, age, and caregiving responsibilities. In Turkey, rural-origin female teachers may face compounded challenges due to limited institutional support and traditional family expectations, whereas urban, single teachers may experience different pressures. This approach reveals how overlapping systems of disadvantage intensify psychosocial stress, emphasizing differentiated vulnerabilities and resources among Turkish female educators (Cho et al., 2013).

3. Psychological resilience

Resilience theory offers a lens to examine how female teachers cope with these layered challenges. Resilience is conceptualized not as a fixed trait but as a dynamic process influenced by internal capacities (problem-solving, emotional regulation), social support (family, colleagues), and institutional conditions (flexible school policies, access to care services) (Masten, 2001; Luthar and Cicchetti, 2000). Within the Turkish education system, resilience allows women to navigate structural constraints, employ coping strategies, and leverage available resources, highlighting their agency despite systemic pressures.

4. Integrative value and study objectives

By combining these three theoretical perspectives, the framework provides a comprehensive lens through which to address the study’s central research question: *“How do female teachers working in Türkiye experience and interpret the effects of work–family conflicts on their psycho-social health within the framework of gender norms, resilience, and multiple identities?”*

This integrative approach moves systematically through the analytical process: Gender performativity and hegemonic masculinity provide the lens to analyze how structural norms create the “double burden” and identify the root structural pressures. Intersectionality then allows for the examination of how these pressures are differentiated across various identity axes such as marital status, age, school type, and caregiving responsibilities, highlighting the varied manifestations of work–family conflict. Finally, resilience theory illuminates the mechanisms through which teachers cope with, adapt to, and resist these structurally imposed challenges, revealing their agency.

In the Turkish socio-cultural context, where traditional gender norms, unequal domestic expectations, and institutionalized hierarchies persist, this integrated framework enables a nuanced understanding of how systemic structures shape female teachers’ psycho-social experiences. Thus, the framework progresses logically from identifying macro-level structural pressures (gender theories), to understanding their meso- and micro-level manifestations (intersectionality), and finally to exploring individual and collective responses (resilience).

## Research aim

The primary aim of this study is to gain an in-depth understanding of the psycho-social health experiences of female teachers working in Türkiye as they navigate the demands of work and family life. Grounded in feminist theory, gender role theory, and resilience frameworks, this research explores how female teachers’ experiences are shaped by socially constructed gender norms, emotional labor expectations, and the intersection of their professional and domestic identities. Through this theoretical lens, the study seeks to reveal how women develop coping strategies, maintain resilience, and negotiate their psycho-social well-being within institutional and cultural constraints. Employing a qualitative phenomenological approach, it examines the dynamic processes through which these teachers interpret and manage work–family conflict while sustaining their professional identities. By linking these findings to theoretical concepts of gendered labor and resilience, the study aims to contribute to educational policies and school management practices that promote equity, mental well-being, and gender-sensitive support systems for female educators. The central research question guiding this inquiry is: “How do female teachers working in Türkiye experience and interpret the effects of work–family conflicts on their psycho-social health within the framework of gender norms, resilience, and multiple identities?”

## Methods

### Research design

This study employs a feminist phenomenological design to explore the psycho-social health experiences of female teachers navigating work–family tensions. Phenomenology, as a qualitative approach, aims to uncover individuals’ lived experiences and the meanings they attribute to them (Moustakas, 1994; Creswell and Poth, 2018). In this study, phenomenology is explicitly grounded in a feminist epistemological stance, treating women’s lived experiences as legitimate and critical sources of knowledge. This approach resonates with Bell hooks' (2000) call to move marginalized experiences from the periphery to the center of theoretical inquiry, thereby challenging dominant patriarchal narratives that often render women’s invisible labor and psycho-social struggles unseen.

The design draws on Husserl’s (1970) descriptive phenomenology and Heidegger’s (1962) existential phenomenology, while integrating feminist critiques that emphasize contextual, gendered, and power-laden dimensions of lived realities. Building on Butler’s (1990) performativity and Crenshaw’s (1989) intersectionality, women’s voices are analyzed as embodied, socially constructed, and discursively mediated experiences. This positioning acknowledges that lived experience is both phenomenological (rooted in subjectivity and meaning-making) and post-structural (shaped through discourse, norms, and institutional power).

The study applies Interpretative Phenomenological Analysis (IPA) (Eatough and Smith, 2017), well suited for exploring meaning-making while accommodating the researcher’s interpretative role. IPA maintains phenomenology’s commitment to understanding subjective experience and allows integration with feminist and post-structural epistemologies.

To ensure credibility and trustworthiness, participants’ preconceptions were bracketed during in-depth, semi-structured interviews, and the researcher engaged in reflexivity throughout the process, acknowledging how personal positionality, gendered assumptions, and power dynamics could influence interpretations.

This feminist phenomenological design enables a nuanced exploration of how socially constructed phenomena such as gender roles, work–family conflict, and psycho-social health are lived and negotiated by women teachers. By integrating descriptive phenomenology, interpretative analysis, and feminist/post-structural critique, the study produces context-sensitive, transformative knowledge, aimed at challenging dominant assumptions and contributing to gender-equitable educational and psychological scholarship.

Note on Data Saturation: Participant selection continued until thematic data saturation was reached, ensuring that the findings reflect comprehensive patterns of experience rather than a predetermined sample size.

### Participants and sampling method

The participants of this study comprised female teachers working at various educational levels across Mardin, Turkey. Purposeful, maximum variation sampling was employed to select information-rich participants who could provide diverse perspectives on the research questions (Patton, 2014; Creswell, 2021). Socio-demographic diversity, including age, marital status, number of children, and professional seniority, was considered to explore how multiple social identities intersect with psycho-social experiences, in line with intersectionality theory (Crenshaw, 1989).

The study included teachers from four main school types—preschool, primary, secondary, and high school—to allow a comparative examination of experiences across educational stages. Selection criteria were as follows:

1) Minimum of three years of teaching experience,
2) Currently active in public schools,
3) Willingness to share perspectives on work–family balance.

Participant recruitment continued until thematic data saturation was achieved, meaning that no new codes or themes emerged during successive interviews and the existing data sufficiently captured the range of experiences relevant to the research questions. This ensured that the sample size was determined by conceptual completeness rather than numerical targets. Thematic saturation enhanced the credibility, trustworthiness, and depth of qualitative findings by reflecting comprehensive and recurrent patterns in participants’ narratives.

### Data collection instruments and procedure

In this study, semi-structured individual interviews were employed as the primary data collection method to explore and gain insight into female teachers’ psycho-social health experiences between their work and family lives. Semi-structured interviews provide a flexible framework that guides the research questions while allowing participants to freely express their experiences and meaning-making processes (Kvale and Brinkmann, 2009). This method is particularly suitable for phenomenological research, as it facilitates the exploration of participants’ personal, contextual, and emotional experiences related to work–family conflict (Seidman, 2006; Van Manen, 2016).

The interview protocol was developed based on themes derived from similar qualitative studies in the literature and aligned with the study’s theoretical framework encompassing gender theory, intersectionality, and resilience. Core thematic questions focused on:

1) How participants define and experience their work and family roles,
2) The primary internal and external stressors affecting their psycho-social health,
3) Challenging situations encountered during work–family conflict,
4) Individual and social coping strategies developed to manage such conflicts.

Interviews were conducted one-on-one, either face-to-face or via online platforms depending on participant preference, each lasting approximately 45–60 min. Written and verbal informed consent was obtained from all participants in accordance with ethical committee standards, ensuring confidentiality, voluntary participation, and psychological comfort throughout the data collection process (Creswell and Poth, 2018).

All interviews were audio-recorded and transcribed verbatim by the researcher. Transcripts were carefully edited to preserve participants’ expressions, and member checking was conducted to enhance credibility and trustworthiness of the data (Lincoln and Guba, 1985). Interviews were carried out in psychologically safe and comfortable environments, supporting participants’ openness and emotional well-being. Additionally, trust was prioritized, and non-directive, open-ended questions were used (Patton, 2014).

This approach ensured the collection of holistic, context-sensitive data reflecting both participants’ lived experiences and their perceptions of the broader social and institutional context.

### Data analysis

The qualitative data obtained in this study were analyzed following Moustakas’s (1994) phenomenological analysis approach, conducted in four key stages:

1) Epoché: The researcher consciously suspended personal biases to focus on participants’ experiences without prejudice.
2) Identification of significant statements: Interview transcripts were examined in detail to identify meaningful statements reflecting participants’ psycho-social health experiences.
3) Development of themes: Significant statements were grouped based on content similarities, generating themes such as work–family conflict, double burden, gender roles, and coping strategies.
4) Synthesis of essence: Identified themes were integrated into a holistic structure to reveal the essence common to participants’ lived experiences.

To enhance credibility, trustworthiness, and confirmability, the findings were supported by member checking with participants (Lincoln and Guba, 1985). Reflexivity was maintained throughout the analysis, acknowledging the researcher’s positionality and interpretative role. These practices ensured that the interpretations authentically represented participants’ experiences and maintained rigor within the qualitative paradigm.

### Trustworthiness and quality criteria

In qualitative phenomenological research, the aim is not to establish positivist validity and reliability, but rather to ensure trustworthiness of the findings through rigor, reflexivity, and transparency (Lincoln and Guba, 1985). This study employed multiple strategies to enhance credibility, dependability, confirmability, and transferability of the research process and outcomes.

Credibility was enhanced through member checking, where emerging themes and interpretations were shared with participants to verify that the analysis accurately reflected their lived experiences. Additionally, data triangulation was applied by recruiting participants from different educational levels, allowing for contextual comparisons and strengthening the authenticity of findings.

Dependability was supported by maintaining detailed records of interviews, coding decisions, and analytical memos throughout the research process. This transparent documentation provides a clear audit trail of how data were generated, coded, and interpreted.

Confirmability was ensured through reflexivity, with the researcher keeping a reflexive journal to critically examine how personal assumptions, positionality, and gendered perspectives could influence interpretations. This practice minimized researcher bias and reinforced the integrity of the findings.

Transferability was facilitated by thick description, situating participants’ narratives within their broader social and institutional contexts. Providing detailed contextual accounts enables readers to assess the relevance and applicability of the findings to other educational and cultural settings.

Taken together, these strategies ensure that the study’s findings are trustworthy, context-sensitive, and epistemologically consistent with a feminist phenomenological approach.

### Ethical approval

This study was conducted following approval from the Ethics Committee of xxx University (Approval Date: 17 April 2024; Document No: 4). All participants were fully informed about the study’s purpose, scope, and the voluntary nature of their participation. Written informed consent was obtained prior to data collection. Participant confidentiality was strictly maintained, with all personal identifiers anonymized. The study procedures adhered to established ethical guidelines for research involving human subjects, including the principles outlined in the WMA Declaration of Helsinki (World Medical Association, 2013).

### Findings

In this section, the qualitative data related to the psychosocial health experiences of female teachers navigating the demands of work and family life are presented thematically, guided by the study’s theoretical framework and research questions. The themes derived from participants’ narratives reveal a rich and multifaceted picture encompassing work-family balance, the impact on psychosocial health, coping strategies, and the influential role of gender norms in shaping these experiences.

Participants’ responses to the research question, “*How do you define the balance between your work and family life?*” are summarized in Table 2.

**Table 2:** Female teachers’ experiences of work-family balance.

Theme	Subtheme	Preschool	Primary	Middle	Secondary	Frequency (f)
Work-family balance	Difficulty in maintaining balance	4	4	5	5	18
	Time management	5	4	3	3	15

The findings reveal that many female teachers experience significant challenges in balancing professional responsibilities with family obligations. Participants frequently described a persistent sense of being pulled in multiple directions, often feeling mentally divided between work tasks and domestic duties. A secondary school teacher articulated this tension vividly: “Thinking about house chores while working makes me feel constantly divided” (P7, Secondary Education). This experience reflects the time-based conflict described by Greenhaus and Beutell (1985) and resonates with Hochschild’s (1989) concept of the “second shift,” highlighting the invisible labor women undertake after paid working hours.

Time management emerged as another critical challenge, particularly among preschool and primary school teachers. Many participants emphasized the difficulty of allocating sufficient time to both work and family responsibilities within a single day, expressing feelings of being constantly rushed or stretched thin. One primary school teacher described this struggle as: “It’s hard to plan my time; I feel squeezed between work and family” (P3, Primary School). From the perspective of Butler’s (1990) performative gender theory, the pressure to simultaneously embody the roles of a “good teacher” and the “ideal mother and spouse” intensifies stress related to daily time management.

The nature of these challenges appeared to vary according to educational level. Teachers in secondary and middle schools highlighted difficulties in maintaining balance due to heavy academic and administrative responsibilities, whereas those in preschool and primary schools emphasized managing time effectively amid individualized student attention and emotional labor.

Overall, the findings indicate that work-family challenges for female teachers are influenced by both structural and cultural factors. Gender norms, institutional expectations, and professional responsibilities collectively shape their experiences, underscoring the need for gender-sensitive policies and professional support mechanisms that address both individual coping and systemic constraints.

Participants’ responses to the question, “*What are the most significant challenges you face between your work and family roles?*” are presented in Table 3.

**Table 3:** Subthemes of role conflict, distribution by school type, and participant statements.

Theme	Subtheme	Preschool	Primary	Middle	Secondary	Frequency (f)
Role conflict	Double burden and invisible labor	5	5	5	5	20
	Psycho-social pressure	4	3	5	4	16

The findings indicate that female teachers frequently experience intense role conflict, arising from the simultaneous demands of professional and domestic responsibilities. Many participants described returning home after a long day at school only to continue fulfilling duties related to motherhood and household management. A middle school teacher articulated this experience: “Work at school is intense, but when I get home, my motherly duties do not end” (K12, Middle School). This account strongly resonates with Hochschild’s (1989) “second shift” concept, illustrating how women’s paid work responsibilities merge with invisible domestic labor.

Participants also reported considerable psycho-social pressure. The expectation to excel both professionally and domestically created persistent stress and fatigue. A high school teacher explained: “I have to be successful both at work and at home all the time, and this exhausts me” (K23, High School). These experiences align with Butler’s (1990) performative gender theory, showing how women continuously enact societal expectations, and Connell’s (2013) hegemonic masculinity, highlighting how patriarchal structures normalize sacrifices demanded from women.

Differences across educational levels were observed, with middle and high school teachers describing the most intense role conflict and psycho-social pressure. The combination of heavy academic and administrative responsibilities with gendered societal expectations contributes to heightened psychological strain, illustrating that work-family tensions are shaped by both structural and cultural factors.

Overall, the findings underscore that female teachers’ experiences of role conflict and double burden are deeply intertwined with institutional demands and gendered social norms, reinforcing the need for gender-sensitive policies and supportive professional frameworks.

Participants’ responses to the research question, “*What do you think about the effects of these challenges on your psycho-social health?*” are presented in Table 4.

**Table 4:** Psycho-social health theme: stress, burnout, and guilt feelings—distribution by school type and participant quotes.

Theme	Subtheme	Preschool	Primary	Middle	Secondary	Frequency (f)
Psycho-social health	Stress and burnout	7	4	6	5	22
	Guilt and anxiety	3	3	4	3	13

The findings reveal that female teachers frequently experience stress and burnout as a consequence of managing simultaneous professional and family responsibilities. Many participants described a growing sense of emotional exhaustion linked to their heavy workload. A secondary education teacher expressed this clearly: “Due to the workload, I sometimes feel exhausted” (K18, Secondary Education). This experience aligns with Maslach and Leiter’s (2016) burnout model, demonstrating that work–family conflict is closely tied to emotional exhaustion in female educators. The particular vulnerability of secondary school teachers to burnout, as highlighted in systematic reviews (García-Carmona et al., 2019), resonates with our findings showing middle and secondary school teachers reporting more intense psycho-social pressure.

Feelings of guilt and anxiety also emerged as prominent aspects of psycho-social health. Several participants reported distress stemming from not being able to dedicate sufficient time and emotional attention to their families. A primary school teacher described this experience: “Not being able to spend enough time with my family makes me very sad” (K5, Primary School). These accounts resonate with Eagly’s (2013) social role theory, highlighting that women’s caregiving and emotional support roles are socially constructed and that unmet expectations can intensify internalized guilt and anxiety.

Variations were observed according to school type. Teachers in preschool and primary schools, who engage in closer and more continuous contact with children, reported higher emotional exhaustion, whereas those in secondary and middle schools emphasized stress related to academic and administrative demands. These differences indicate that psycho-social health experiences are shaped by both professional domains and the nature of teacher-student interactions.

Overall, the findings suggest that female teachers’ psycho-social well-being is influenced by the interplay of professional demands, family responsibilities, and societal expectations, highlighting the need for supportive institutional policies and practices that address both individual coping and systemic pressures.

Participants’ responses to the question, “*What resources or strategies support you in balancing your work and family life?*” are presented in Table 5.

**Table 5:** Coping strategies.

Theme	Subtheme	Preschool	Primary	Middle	Secondary	Frequency (f)
Coping strategies	Social support	4	4	5	5	18
	Personal resilience	3	4	3	5	15

The findings reveal that female teachers commonly employ social support and personal resilience as strategies to manage psycho-social pressures arising from work and family responsibilities. Participants frequently emphasized that emotional and instrumental support from family members, spouses, colleagues, and friends helped them navigate challenges in both domains. A middle school teacher described this experience: “Even though my job is very demanding, feeling the support of my colleagues empowers me. Talking with people who go through the same experiences is very helpful” (K13, Middle School). Similarly, a preschool teacher highlighted the role of family support: “My spouse helps a lot with cooking and taking care of the children. This allows me to catch my breath” (K2, Secondary Education). These accounts illustrate the protective role of social networks in coping, consistent with Masten’s (2001) psychological resilience model and Luthar and Cicchetti’s (2000) concept of protective environmental factors.

Participants also reported engaging in personal resilience strategies to sustain their well-being. These included self-care routines, time management, pursuing hobbies, and setting emotional boundaries. One primary school teacher explained: “I have to make time for myself. Going for a walk on weekends, reading a book, or just staying quiet refreshes me” (K8, Primary School). Another secondary education teacher emphasized internal motivation: “What keeps me going is my inner motivation. No matter how tired I am, my commitment to my profession motivates me” (K24, Secondary Education). These narratives demonstrate how women mobilize personal resources to regain balance after periods of stress, aligning with Richardson et al.’s (1990) resilience framework.

Coping strategies were also interpreted through a gendered lens. The use of social support and personal resilience can be seen as forms of resistance against societal expectations of “ideal womanhood” (Butler, 1990). Furthermore, variations in coping approaches reflect intersectional differences, as women navigate the simultaneous demands of professional, familial, and social identities (Crenshaw, 1989).

Across educational levels, both social support and personal resilience were commonly employed, though teachers in middle and secondary schools particularly emphasized the importance of social support networks, likely due to the increased professional and bureaucratic demands at these levels.

Overall, these findings underscore that female teachers actively engage in both individual and collective coping mechanisms, highlighting the interplay of personal agency, social resources, and structural factors in maintaining psycho-social well-being.

Participants’ responses to the research question, “*How do societal gender roles, as a woman, affect your work and family life?*” are presented in Table 6.

**Table 6:** Gender-related themes and distribution by school type.

Theme	Subtheme	Preschool	Primary	Middle	Secondary	Frequency (f)
Gender roles	Traditional roles and expectations	6	4	6	4	20
	Guilt and pressure	4	3	4	3	14

The findings show that domestic responsibilities continue to fall predominantly on women, reflecting the persistence of gendered social norms. Many participants described that in addition to their professional duties, they bear the bulk of household chores, childcare, and emotional support responsibilities. A secondary school teacher expressed this vividly: “Because we are women, household chores are always our responsibility. When I come home in the evening, the cooking, laundry, children… all are waiting for me” (K26, Secondary School). This experience exemplifies Hochschild and Machung’s (2012) “second shift,” highlighting the invisible labor women carry after paid work, which contributes to physical and psychological exhaustion.

The gendered division of labor also intersects with broader societal expectations, generating feelings of inadequacy and guilt. A middle school teacher articulated this pressure: “Society expects us to be perfect in every area. You must be a good teacher, a good spouse, and a good mother. When I fail, I feel guilty” (K14, Middle School). These emotional responses align with Butler’s (1990) performative gender theory, which suggests that women are compelled to continuously enact idealized femininity in both public and private spheres. The phenomenon can also be interpreted through Crenshaw’s (1989) intersectionality framework, showing how pressures intensify at the intersections of gender, motherhood, marital status, and professional identity.

Although the experience of gender-based pressures was reported across all school types, it was particularly emphasized by preschool and middle school teachers. This may reflect the higher pedagogical and emotional demands associated with younger students, as well as the prevalence of younger mothers in these teaching roles, which heightens the potential for role strain between professional and family obligations.

Overall, these findings demonstrate that female teachers’ domestic burdens are structurally and socially mediated, intersecting with professional responsibilities and societal expectations. They underscore the need for policies and institutional practices that acknowledge and redistribute unpaid labor and provide supportive mechanisms to mitigate psycho-social stress.

Participants’ responses to the research question, “*What strategies do you employ to maintain your psychosocial well-being?*” are presented in Table 7.

**Table 7:** Protective strategies and their distribution by school type.

Theme	Subtheme	Preschool	Primary	Middle	Secondary	Frequency (f)
Protective strategies	Personal care and hobbies	5	4	4	4	17
	Social support	4	3	5	3	15

The findings indicate that female teachers consciously employ coping strategies to protect their psychosocial health from the pressures of balancing work and family life. Two main approaches emerged: personal care and hobbies, and social support (Table 8).

**Table 8:** Literature-based prioritization on work–family conflict and psychosocial health.

Theme	Subtheme	Literature connection	Priority school levels
Work–family conflict	Time management and workload	Greenhaus and Beutell (1985)	Secondary school > Middle school
Role conflict and double burden	Domestic responsibilities	Hochschild (1989)	Secondary school > Primary school
Psycho-social health effects	Burnout, stress	Maslach and Leiter (2016)	Secondary school > Middle school
Gender roles	Feelings of guilt	Eagly (2013)	All school types, especially secondary school
Coping strategies	Social support, resilience	Masten (2001)	All school types

Many participants highlighted the importance of personal relaxation and hobbies in mitigating stress. For instance, one teacher shared, “Listening to music and taking walks helps me feel better” (Primary School). Such strategies reflect the individual resilience processes emphasized by Masten (2001) and Richardson et al. (1990), illustrating how teachers actively regulate their emotions and maintain psychological equilibrium in response to professional and domestic demands. Across all educational levels, teachers engaged in similar activities to sustain personal well-being, although those working in secondary schools reported slightly greater reliance on structured personal care due to higher academic and administrative responsibilities.

In addition to individual strategies, social support emerged as a crucial coping mechanism. Participants described the protective role of family, colleagues, and friends in buffering psychosocial stress. One secondary school teacher explained, “Spending time with my family and friends lifts my morale.” This observation aligns with Luthar and Cicchetti’s (2000) concept of protective environmental factors, highlighting how social networks serve as a key resource for resilience. Secondary school teachers, in particular, emphasized social support, reflecting the greater psycho-emotional load associated with working with adolescent students and managing more complex institutional responsibilities.

These findings demonstrate that female teachers’ coping strategies are both individual and socially mediated, varying according to professional demands and educational context. By situating the coping mechanisms within resilience theory and emphasizing the interplay between personal agency and social resources, the study underscores the need for school-based psychosocial support programs that are sensitive to the diverse requirements of teachers across different educational stages.

This study examined the psychosocial challenges and coping strategies experienced by female teachers in balancing their work and family lives within a school-based context. Based on the literature and empirical data, five key themes emerged. The first theme is work–family conflict; guided by Greenhaus and Beutell’s (1985) time management theory, especially secondary and middle school teachers struggle to balance professional demands and school responsibilities with family duties. The second theme encompasses role conflict and the double burden concepts articulated by Hochschild (1989), highlighting the psychosocial impact of the invisible labor women undertake both at school and at home. The third theme relates to feelings of burnout stemming from intense occupational demands, consistent with the burnout and stress theories proposed by Maslach and Leiter (2016). The fourth theme addresses the guilt experienced within the school environment through the lens of Eagly’s (2013) social role theory on gender expectations. Finally, social support and resilience mechanisms, as conceptualized by Masten’s (2001) resilience theory, play a crucial role in protecting female teachers’ psychosocial health. The findings indicate that work-family balance is shaped by the interplay of institutional demands and prevailing gender norms.

## Discussion and conclusion

This study employed a feminist phenomenological design to explore the psycho-social health experiences of female teachers in Turkey as they negotiate work and family life. The findings indicate that women’s well-being is shaped not only by individual coping strategies but also by the structural reproduction of gender norms within educational and domestic spheres, as highlighted by gender performativity theory (Butler, 1990) and hegemonic masculinity (Connell, 2013). Participants’ experiences of guilt, stress, and burnout reflect internalized societal expectations of the “ideal mother” and “ideal worker,” showing how personal struggles are embedded in broader patriarchal structures.

Furthermore, the pressure to perform flawlessly in both domains may extend to the professional arena, potentially impacting women’s leadership trajectories. The added scrutiny and pressure that often accompany women in leadership positions can contribute to their shortened longevity in such roles (Dwivedi et al., 2021). This suggests that the barriers faced by female teachers are not confined to work-family balance but can also hinder their career advancement into administrative and leadership positions within the education system.

An intersectional lens (Crenshaw, 1989) reveals that these struggles are not uniform. Teachers’ experiences varied by motherhood status, socioeconomic background, and professional seniority, demonstrating how intersecting identities intensify or mitigate psycho-social burdens. For instance, participants with multiple caregiving responsibilities reported more pronounced exhaustion than unmarried or childless colleagues. These findings underscore the importance of policies that account for diverse experiences rather than adopting one-size-fits-all approaches.

The findings also illuminate the role of resilience theory (Masten, 2001) in understanding women’s agency. Participants employed adaptive strategies such as mobilizing social support and engaging in self-care. However, resilience alone is insufficient when structural inequalities remain, consistent with Connell and Pearse’s (2015) critique. This integration of resilience with structural analysis highlights that interventions must address both individual coping and systemic transformation.

By weaving feminist epistemology throughout, this research centers women’s voices as legitimate sources of knowledge and demonstrates how institutional and cultural norms reproduce gendered burdens. This methodological stance echoes Bell hooks' (2000) imperative to center marginalized experiences as a form of resistance against patriarchal domination. The integration of gender theory, intersectionality, and resilience allows a holistic interpretation: personal experiences of stress and coping are inseparable from broader societal structures and cultural expectations.

Policy and practice implications: Supporting female teachers requires multi-layered interventions. Educational authorities should implement flexible work arrangements, expand psycho-social support mechanisms, and develop gender-sensitive professional development programs. Such institutional reforms align with global recommendations for gender-responsive social policies in education that address the specific needs and constraints facing female educators (Subrahmanian, 2011). Societal change—including public awareness campaigns and community-based initiatives—is essential to dismantle patriarchal structures perpetuating work–family conflict.

Conclusion: This study reveals that the psycho-social health experiences of female teachers are not merely individual phenomena but are also shaped by structural inequalities rooted in gender norms. The findings indicate that women experience guilt, burnout and emotional strain as they become trapped between the conflicting expectations of being the “ideal mother” and the “ideal employee.” At the same time, their development of resilience and social support strategies can be interpreted as a form of silent resistance against these pressures. In conclusion, promoting the well-being of female teachers requires urgent structural and cultural transformations within educational institutions that prioritize gender equality, rather than relying solely on individual coping efforts.

### Limitations of the study

This study explored the psycho-social health experiences of female teachers in their work and family lives through a qualitative phenomenological approach. However, the findings are limited to specific regions and types of schools, and thus cannot be generalized. The use of purposive sampling and a small sample size may not fully represent the broader population of female teachers. Data collection relied solely on in-depth interviews, which limits data triangulation. Additionally, participants’ responses may have been influenced by social desirability bias. These limitations nonetheless provide valuable opportunities for future research employing diverse samples and mixed or alternative methodologies.

### Recommendations

This study has identified key factors affecting the psycho-social well-being of female teachers, highlighting the critical roles of work-family balance, social support systems, and gender roles. Based on the findings, the following recommendations are offered for policymakers—particularly the Ministry of National Education (MoNE)—and future researchers. The following recommendations draw on both our empirical findings and established frameworks for gender-sensitive policy in education (Subrahmanian, 2011):

1. Development of institutional flexibility and support policies

MoNE should implement flexible work arrangements such as adjustable working hours, part-time employment, and extended leave policies to alleviate work–family conflict among female teachers. Given the particularly high workload at the secondary school level, such measures are essential for sustaining professional well-being. This recommendation is grounded in Greenhaus and Beutell’s (1985) work–family conflict model and supported by flexible work practices observed in OECD countries.

2. Expansion of psychosocial support and counseling services

In line with Maslach and Leiter’s (2016) burnout theory, psychological counseling and guidance services should be strengthened to mitigate the emotional burdens faced by teachers. In-service training programs should focus on stress management and resilience-building strategies. Drawing on Masten’s (2001) resilience model, protective social support networks should be established within educational institutions.

3. Enhancement of gender equality and awareness education

Based on Butler’s (1990) theory of gender performativity and Connell’s (2013) concept of hegemonic masculinity, gender modules should be integrated into both pre-service teacher education and in-service training programs. Such interventions can raise awareness of the often-invisible burdens placed on women through expectations of the “ideal mother/wife/teacher” and promote a more equitable distribution of responsibilities.

4. Support for career advancement and leadership opportunities

From the perspective of gender equality as emphasized by Connell and Pearse (2015), programs such as mentorship, leadership training, and career planning support should be developed to facilitate women’s access to administrative positions. These initiatives would not only enhance female teachers’ professional autonomy but also contribute to institutional resilience.

5. Guidance for future research

Further mixed-method studies should explore the psycho-social experiences of female teachers across broader, more diverse samples—especially within different geographical, cultural, and socio-economic contexts. Comparative studies with male teachers are also needed to map gender-based disparities. Additionally, the impact of post-pandemic digitalization on female teachers should be critically examined.

## Data Availability

The original contributions presented in the study are included in the article/supplementary material, further inquiries can be directed to the corresponding author/s.
